# Screening for Early Emerging Mental Experiences (SEE ME): A Model to Improve Early Detection of Psychosis in Integrated Primary Care

**DOI:** 10.3389/fped.2022.899653

**Published:** 2022-06-10

**Authors:** Kristen A. Woodberry, Kelsey A. Johnson, Lydia A. Shrier

**Affiliations:** ^1^Center for Psychiatric Research, MaineHealth, Portland, ME, United States; ^2^Department of Psychiatry, Tufts School of Medicine, Boston, MA, United States; ^3^Department of Psychiatry, Beth Israel Deaconess Medical Center, Boston, MA, United States; ^4^Division of Adolescent and Young Adult Medicine, Boston Children’s Hospital, Boston, MA, United States; ^5^Department of Pediatrics, Harvard Medical School, Boston, MA, United States

**Keywords:** first episode psychosis (FEP), early intervention (EI), prodrome, serious mental illness (SMI), adolescents

## Abstract

Early intervention in serious mental health conditions relies on the accurate identification of adolescents and young adults at high risk or with very recent onset of psychosis. Current early detection strategies have had limited success, identifying only a fraction of these individuals within the recommended 3- to 6-month window. Broader public health strategies such as population screening are hampered by low base rates and poor self-report screen specificity. Screening for Early Emerging Mental Experiences (SEE ME) is a three-stage “SCREEN—TRIAGE—ENGAGE” model for the early detection of psychosis in integrated primary care adolescent and young adult patients during the period of peak onset. It builds on the KNOW THE SIGNS—FIND THE WORDS—MAKE THE CONNECTION framework outlined on psychosisscreening.org and developed with input from community collaborators. Systematic screening aims to expand the reach of early detection and reduce reliance on provider knowledge. Triage and engagement by trained mental health clinicians aims to improve the specificity of screen responses, enhance engagement in appropriate care, and reduce provider burden. Leveraging the low stigma of primary care, its reach to non-help-seeking adolescents and young adults, and the mental health training of clinicians within integrated care practices, SEE ME has potential to improve the benefit/risk ratio of early detection of psychosis by improving both the sensitivity and specificity of screening and clinical response. We review the rationale and design of this promising model.

## Introduction

Psychotic symptoms are widely considered a marker of the most serious mental health conditions, and predictive of more severe outcomes (1, 2). The first three-to-six months after onset of acute psychosis is a critical window for early intervention in these conditions (1, 3–5). It is the best opportunity to improve outcomes for affected youth and mitigate the elevated risks for suicide, hospitalization, and violence that peak with the emergence of acute psychosis (6–8). A longer *duration of untreated psychosis* (DUP) is robustly associated with greater symptom severity and functional impairment, even long-term (3, 4, 9). Most individuals do not receive appropriate care until well beyond this window (5, 9).

Factors contributing to DUP have been identified primarily through retrospective interviews with individuals once they have received *coordinated specialty care* (CSC, current best practice for psychosis). A prominent conceptualization views these factors within a “supply and demand” framework (10). “Supply” factors are those that influence referral pathways and access to treatment. They include aversive experiences with the mental health system, provider misattribution, and racial disparities (11, 12). “Demand” factors are those that influence help-seeking or treatment engagement. These include concerns about stigma, overvaluing of self-sufficiency, lack of social support, and misattribution of symptoms (10, 11). Access to specialty psychosis treatment, usually *via* hospitalization, often involves coercion and other aversive experiences, such as restraint, that impede treatment engagement and future help-seeking (13, 14). Strategies that foster decisional autonomy or shared decision-making must become more efficient to simultaneously reduce DUP and effectively engage individuals in a pathway to recovery (9, 14, 15).

New efforts must extend beyond the current reliance on community education and treatment of help-seeking individuals (2, 5). Screening is a logical next step but common mental health screens, used only sporadically, do not include items probing psychosis. It is impractical to conduct widespread specialized interviews, yet none of the internationally-developed self-report psychosis screening tools (see section “Screening Tool Options”) are sufficiently accurate in general population, particularly adolescent, samples (16, 17). Screens for less common conditions such as schizophrenia generally identify too many “false positives” (18). Screening for psychosis poses additional challenges. Psychotic-spectrum experiences (e.g., hearing a voice when no one is present, suspiciousness), are simultaneously more common and less predictive of later psychotic disorder in children and adolescents relative to adults (19, 20). Abstract and highly subjective psychosis probes are easily misunderstood by individuals with low cognitive and language capacities, but this misunderstanding is only identified through interview queries (21). Finally, some psychosis screen items, e.g., those probing supernatural experiences, suspicion, or paranoia, may be “falsely” endorsed by individuals with certain religious beliefs (e.g., ability to hear God’s voice) or exposure to crime or discrimination (i.e., justified fears or suspiciousness). Misattribution of these endorsements to psychosis can exacerbate existing health disparities (16, 22, 23). Screening by itself may not have a positive benefit-risk ratio (2). Early detection strategies are needed that identify many more youth within the critical window for intervention, helping them stave off aversive interactions with mental health and other systems in response to crises, while simultaneously minimizing risks to individuals at low risk for psychosis.

## See Me Model

### Overview

The authors propose the SEE ME model based on extensive discussions with New England primary care, psychosis specialty care, and community stakeholders. Its three stages, or components, were conceptualized to improve reach (sensitivity) and timeliness of early detection of serious mental health conditions in a manner that minimizes psychosis-specific treatment/labeling exposure for non-psychotic or subthreshold experiences (improved specificity). Importantly, it was designed with the aspirational goal of averting crises, hospitalizations, suicide attempts, violence, or police involvement commonly experienced by current youth. For this and other practical reasons (see section “Focus on Primary Care”), it was designed for United States primary healthcare clinics that serve the general community and have the integrated mental health capacity to conduct the 3 component activities: screen, triage, and engage.

**FIGURE 1:**
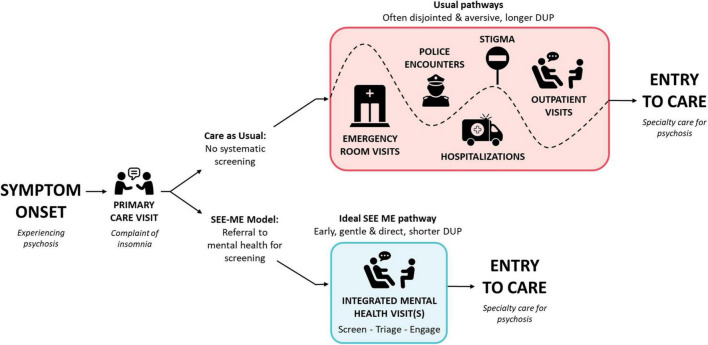
Targeted impact of SEE ME on pathways to care. Since SEE ME seeks to identify acute psychosis as well as high psychosis risk, the time from psychosis onset to specialty care represents the Duration of Untreated Psychosis (DUP) for individuals with psychotic disorders and Duration of Untreated Illness (DUI) for individuals identified during an at risk state. Those at risk who transition to a psychotic disorder would have a subsequent DUP.

The underlying assumption of SEE ME is that individuals suffering with psychosis are not “seen” in time. There are too few healthcare contacts that explicitly invite disclosure and explore private experiences of altered reality in a non-stigmatizing manner. When community primary and mental healthcare providers do recognize psychosis, many are unsure how to respond or connect individuals with timely care (24, 25). Figure 1 illustrates two pathways to care for a hypothetical individual presenting to primary care with insomnia, an early, but non-specific, symptom commonly experienced in psychotic disorders. Usual care (top) too often involves elongated and often aversive pathways from psychosis onset to appropriate care. SEE ME (bottom) is designed to facilitate a shorter, direct, and gentler path.

### Focus on Primary Care

SEE ME’s focus on primary care settings is based on four factors. First, primary care is the natural home for early intervention in potentially chronic conditions; providers are trained to recognize clinical syndromes. Second, primary care settings carry low stigma, particularly for individuals and families who do not identify as having mental health concerns or are reticent to seek mental healthcare (25). Third, primary care settings typically see individuals through adolescence into adulthood, individuals not in school, and individuals who initially present with physical health concerns such as insomnia, inattention, or unexplained concerns about body integrity or functioning. Fourth, by following patients over time, primary care providers have a unique opportunity to note change and develop a relationship that invites disclosure of mental health concerns.

## Stage Specifics

**FIGURE 2:**
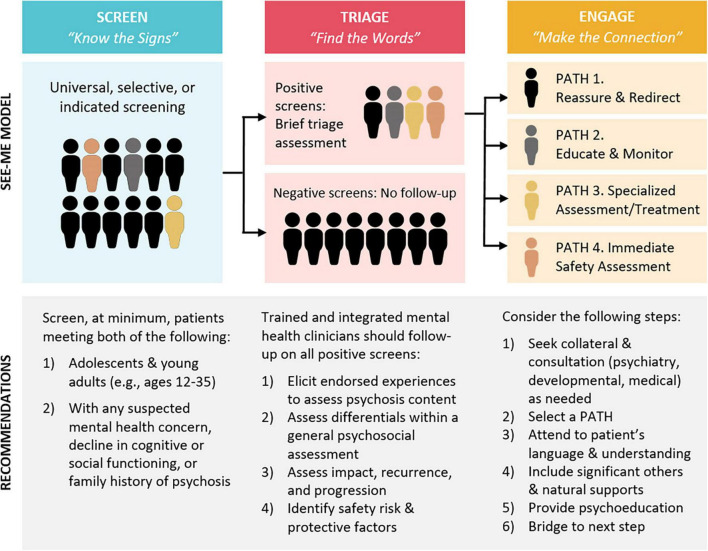
SEE ME model.

Figure 2 illustrates the three stages of the SEE ME model (screening, triage, and engagement), and their linkage to the KNOW THE SIGNS, FIND THE WORDS, and MAKE THE CONNECTION materials available at www.psychosisscreening.org (26). These online materials were developed through iterative discussions with community stakeholders to support indicated screening: provider recognition of warning signs, inquiry about psychosis, and connection with services. However, anecdotal evidence from provider training and widespread resource distribution in Massachusetts suggested that education and resources alone had minimal impact on improving early detection. Similar to other successful early intervention protocols (e.g., Screening, Brief Intervention, and Referral to Treatment, SBIRT) (27), SEE ME proposes systematic screening, i.e., asking all or a subset of patients a minimum set of questions about psychotic-spectrum experiences, ideally *via* self-report. Leveraging the integrated care structure, the bulk of “SCREEN-TRIAGE-ENGAGE” activities are borne by integrated clinicians with mental health expertise. Medical providers can play a role in recognizing mental health concerns, including potential psychosis, and connecting patients to mental health services, but do not carry the primary burden for this. Triage and engagement of positive screens, conducted by a mental health clinician across one or more visits or collateral contacts, should improve outcomes and mitigate risks for both “true” and “false” positives.

We outline the core components and options that may vary with funding and practice characteristics.

### Screening

#### Screening Tool Options

Systematic screening depends on the availability of a very brief self-report measure written at no more than a 5th grade reading level (2). (Ideally, caregiver and interview versions would also be available.) The tool should have established validity for eliciting psychotic-spectrum experiences, be easily scored, and have thresholds and/or norms for diverse populations (and considerations for relevant sociodemographic factors and specific selected or indicated subpopulations). Tools do exist and are used in primary care (e.g., Prodromal Questionnaire Brief, PQ-B), but no single tool meets all of these criteria. The reader is referred to available analyses and guidelines to select the best screen for their context (2, 28–30). The SEE ME model is designed to accommodate current tools and their limitations through skilled mental health triage of all “positive” screens.

#### Universal vs. Selective vs. Indicated Options

The long-term goal is universal screening: screening all, e.g., adolescent and young adult, primary care patients for psychosis. Broad implementation awaits a brief general mental health screening tool that includes items related to psychosis. Selective screening, screening subpopulations with above-average risk (e.g., patients with identified mental health concerns or a positive family history of psychosis) may be most realistic in the short term, particularly if conducted entirely by mental health clinicians. Indicated screening entails screening or assessment of individuals on the basis of symptom or behavioral indicators. This most common strategy relies on both observable indicators and savvy providers. Strategies may also be combined, for instance, mental health clinicians conducting selective screening of all referred patients within a set age range and physicians in the same practice conducting their own indicated screening of patients when they recognize an early warning sign (e.g., new difficulty reading or following a conversation).

#### Protocol Considerations

Success of any screening effort relies on efficient workflows, technical aids and staffing, and clear guidelines for administering, documenting, tracking, and responding to screens. Electronic health record access and entry is ideal for providers, whereas accessibility and privacy are priorities for patients. Protocols must protect adolescent privacy to assure accurate report of potentially sensitive experiences whether *via* paper, tablet, or online. Simultaneously, screen results and triage decisions must be documented to prompt appropriate next steps and allow for monitoring over time.

### Triage

The mental health clinician’s primary task is to follow-up on positive psychosis screens and any reported or observed indicators to determine the appropriate next step. The assessment must be brief. These integrated mental health clinicians do not typically have the time or training to conduct structured diagnostic interviews to differentiate attenuated and acute psychosis from other concerns. For young people who have difficulty sharing their experiences, clinicians may extend their assessment or return to sensitive or vague subjects once a trusting relationship is established.

Maintaining curiosity and closeness to the patient’s experience and language are essential to understanding the nature of these experiences within a broad psychosocial and mental health context. Clinicians must have sufficient diagnostic skills and access to medical, developmental, psychiatric, and other specialty consultations to consider alternative diagnoses and comorbidities (e.g., Trauma-related Disorders, Anxiety and Obsessive Compulsive Disorders, Developmental Disorders). For complex and atypical presentations, referral for comprehensive medical-mental health evaluations is recommended.

The first assessment task following a positive screen or indicated referral is to determine whether there is psychotic content and/or whether experiences or indicators are best explained by cultural, developmental, psychosocial, or other mental health factors. As illustrated within Figure 2, patients without specific psychosis concerns are triaged to appropriate mental health or social services (ENGAGE: PATH 1.). For patients with psychotic-spectrum experiences, there is a second task: to assess whether patients’ experiences rise to the threshold warranting a specialized psychosis assessment (if no, PATH 2; if yes, PATH 3). Clinicians assess for impact, persistence, and progression of psychotic-spectrum experiences, corresponding to established criteria for psychosis risk and disorder (31).

Clinicians must also assess and respond to potential imminent risk for harm to self or other (if yes, PATH 4). This should follow good clinical practice, with clinicians adhering to agency procedures and relevant laws. It is critical that clinicians avoid assuming that psychotic symptoms are by definition dangerous. Risk must be taken seriously, but some patients can live safely with even violent command hallucinations.

### Engagement

#### Critical Aspects of Engagement

Integrated mental health services provide an opportunity to help patients find the language to share their private experiences with caring professionals and embark on pathways toward meaningful lives. Engagement is its own stage in the SEE-ME model to maximize the likelihood they are successful. In addition to general good practice (e.g., understanding and respecting patients’ values and goals), components of engagement specific to psychosis include (1) providing psychoeducation to help patients envision and begin to own their recovery, (2) explicitly countering common psychosis-related myths and stereotypes (e.g., that these experiences are the person’s fault, untreatable, or imply inevitable violence, disability, or inhumanity) and, (3) helping them establish a safe connection with appropriate psychosis-relevant care. Engagement may consist of a brief discussion, introduction, and warm handoff to a program clinician, a gradual relationship-building, targeted psychoeducation and family meetings, shared viewing of online videos and testimonials (e.g., https://strong365.org), handouts or books, or introduction to a peer support partner or group in recovery. With particularly disengaged youth or families, every effort must be made to “leave the door open” and avoid aversive interactions.

For the roughly 5–15% of young people with subthreshold (e.g., non-distressing or infrequent) psychotic-like experiences (20), clinicians “Educate and Monitor” and/or rescreen at regular intervals (e.g., every 3–12 months; PATH 2). This education focuses on the importance of good mental hygiene and of seeking help if experiences progress. Thoughtful safety discussions can be protective beyond physical safety if they address internalized stigma, prompt important environmental changes, and open up productive and compassionate communication with natural supports.

#### Engaging Families and Other Natural Supports

Families and caregivers are often the ones to seek help on behalf of their loved ones. Even those who may minimize or dismiss concern about their loved one’s mental health will often support efforts to foster success with school, work, or financial independence. Adolescent and young adults should be supported in directly sharing their experience with these significant others whenever possible to keep the focus on their perspectives and language and to facilitate effective communication. Psychoeducation provided to a young person and family member together can create a shared understanding, elicit and address misunderstandings and mistaken assumptions, and foster normalizing language to reduce stigma. Individual time can be provided to address sensitive questions, but SEE ME advocates for joint engagement when possible. Family support (e.g., National Alliance on Mental Illness, NAMI) can have a major impact on the young person’s engagement and wellbeing.

#### Engagement Through Messaging and Clinic and Community Cultures

Fostering a practice and community culture that challenges stigma, expects recovery, and normalizes a continuum of mental wellness may improve mental health literacy and more rapid access to quality care. This can occur through waiting room, website and other public messaging, visit protocols, and provider trainings.

### Collaboration and Training

Although SEE ME provides a model, eliciting practice-level expertise, ideas, and concerns is needed to create efficient and effective screening workflows and provider engagement. Identifying and supporting practice champions and offering flexibility is key. A commitment to systematic screening implicitly communicates that psychotic-spectrum concerns are not uncommon, are more easily managed when shared than when kept secret, and that someone is interested in hearing about them. Repeated screening (e.g., annually) may facilitate more rapid disclosure or engagement even for initially reluctant youth or families.

Practice-level trainings should include the voices of lived experience and review of patients not “seen early enough” to help with provider buy-in. Spreading trainings over time facilitates integration and sustained vigilance. Trainings targeting medical providers must improve awareness of warning signs, strengthen provider comfort discussing psychosis, emphasize a low threshold for referral to integrated mental health clinicians, and address common medical and case management challenges. Trainings for the integrated mental health clinicians cover epidemiology and conceptual aspects of psychosis onset to shape realistic expectations and capacity for psychoeducation. Role plays are used to establish competency in specific “SCREEN-TRIAGE-ENGAGE” skills. Practice managers and others who may play a role in administering or tracking self-report screens are trained on the workflow, tone and wording for introducing screens to patients, and strategies to enhance privacy, respond to patient and family questions, and coordinate subsequent referrals.

#### Skills Training

Although some primary care and mental health providers are highly skilled in interviewing adolescents and young adults about mental health concerns, few are skilled in interviewing patients about psychosis. Skills must be taught and practiced before a clinician can be expected to employ them. Skills in talking about psychosis center on creating a shared sense of curiosity and goal for well-being. Medical and mental health clinicians need skills for eliciting and responding to disclosures of psychotic-spectrum experiences, for differentiating psychotic from non-psychotic content, clinical from non-clinical experiences, integrating first person and collateral reports, and on when to seek consultation with specialty services. Clinicians also need skills for motivating or facilitating youth and family curiosity, hope, and engagement in appropriate care.

Regular supervision by psychosis experts, either *ad hoc* or on a monthly basis, is essential to help frontline clinicians ask pivotal triage questions (e.g., to differentiate social anxiety from paranoia or hallucination from traumatic reliving). Supervision can be an essential forum for learning how to engage a diverse spectrum of young people and family members, particularly those who are actively psychotic, have low mental health literacy, have negative perceptions of mental health services, and face multiple or intersectional barriers in accessing specialty care services. Every effort must be made to avoid aversive interactions. Finally, clinicians must learn and practice skills for honoring family values, addressing stigma, and collaborating in the management of safety risks.

## Next Steps for Testing This Model

### Feasibility

Given challenges implementing even well-established mental health screens and universal screening protocols such as Screening, Behavioral Intervention, and Referral to Treatment (SBIRT), assessment of feasibility is an essential first step. We need evidence that integrated care practices will provide the necessary time and infrastructure for training and implementation. Data are needed on screen response patterns, particularly rates of distress due to psychotic-spectrum experiences, for diverse primary care setting populations (pediatric, family medicine, and adult medicine) (30). A feasibility study is needed to provide estimates of time, training, and cost for implementation, and the system supports (e.g., electronic health record tools) needed to assure scalability. Finally, it will be important to estimate the degree to which screening identifies young people whose psychotic experiences are not already known, triage rules out individuals whose positive screens are unrelated to psychosis, and individuals with threshold level symptoms (and their families) can be effectively engaged in specialty care.

### Screening and Triage Accuracy: Will SEE ME Identify the Right Individuals?

In addition to feasibility, success of the model depends on the accuracy of the screening tools and clinician triage benchmarked against structured interview assessment of psychosis risk syndromes and psychotic disorder, including future psychotic disorder. As discussed in section “Screening,” screening “accuracy” will differ according to the screen and threshold used and the ages, clinical needs, and sociodemographic characteristics of the population screened. Accuracy of triage across different levels of prior training and experience as well as demonstrated SEE ME skills will be important for refinement of training and supervision by specialists in psychosis assessment and treatment.

### Impact on Early Detection and Intervention Outcomes

The following measures for SEE ME practices over time and relative to non-SEE ME practices and other agencies are suggested to establish impact:

1) Number and percent of referrals to local Coordinated Specialty Care (CSC) programs.
2) Degree to which CSC referrals reflect the community’s sociodemographic mix.
3) Rates of attendance in CSC intake appointments and subsequent CSC treatment components.
4) Rates of emergency department visits, hospitalizations, suicide attempts, violent incidents, and police encounters prior to and during CSC program participation.
5) DUP and Duration of Untreated Illness (DUI, measured from onset of prodromal symptoms) for different offsets: first psychosis diagnosis, first psychosis- or psychosis-risk-informed therapy, first antipsychotic, and CSC.
6) Short- and long-term clinical and functional outcomes.

Well-controlled randomized trials, clustering at the level of practice or patient, will be needed to separate out the impact of SEE ME from other initiatives with potential impact on results.

## Discussion

In spite of advances in early detection and intervention in serious mental health disorders, most affected individuals, if they receive appropriate care, receive it well outside of the window for best outcomes. SEE ME aims to reduce DUP across broader segments of the United States population with a 3-stage psychosis screening, triage, and engagement model situated within primary care. Leveraging the low stigma environment of this setting, it holds particular promise for reaching individuals who are not seeking mental healthcare or help for psychotic-spectrum concerns. It relies on mental health clinicians integrated within primary care teams to conduct all three stages, leveraging well-placed mental health expertise and minimizing added burden on physicians.

The innovation of this model includes its potential for identifying psychosis and high risk syndromes prior to the mental health crises that typically trigger referral to specialized care and to reach currently underserved populations. Systematic inquiry into psychotic-spectrum symptoms implicitly educates young patients that psychotic-spectrum experiences are not rare and that primary care providers are interested in hearing about these experiences. Careful triage and engagement can improve early detection of subthreshold symptoms that progress to threshold symptoms over time, improve the quality of care for non-psychotic mental health concerns, and directly address internalized stigma within mental health sessions and psychoeducation (32).

Next steps for testing this model include establishing its feasibility, the capacity for clinical triage to improve on the accuracy of screening alone, and the model’s efficacy in reducing the duration of untreated psychosis and adverse events, and engaging currently underserved young people and families in state-of-the-art care. The long term vision is that no young person experiences psychotic-spectrum experiences without someone inviting them to share these experiences and receive help.

## Data Availability Statement

The original contributions presented in this study are included in the article/supplementary material, further inquiries can be directed to the corresponding author.

## Author Contributions

KW initiated and led the conceptualization of the SEE ME model and wrote the initial draft. KJ made substantive contributions to the development of the SEE ME model, created the figures, and edited the manuscript. LS made substantive contributions to the development of the SEE ME model and edited the manuscript. All authors contributed to the article and approved the submitted version.

## Conflict of Interest

The authors declare that the research was conducted in the absence of any commercial or financial relationships that could be construed as a potential conflict of interest.

## Publisher’s Note

All claims expressed in this article are solely those of the authors and do not necessarily represent those of their affiliated organizations, or those of the publisher, the editors and the reviewers. Any product that may be evaluated in this article, or claim that may be made by its manufacturer, is not guaranteed or endorsed by the publisher.
